# Aphthous lesions turned out to be neonatal very early-onset inflammatory bowel disease: a case report

**DOI:** 10.3389/fped.2024.1433852

**Published:** 2024-10-30

**Authors:** Felix Hutmacher, Selina Doerig, Rainer Grobholz, Henrik Köhler, Philipp Meyer, Philipp Baumann

**Affiliations:** ^1^ Department of Neonatology, Kantonsspital Aarau, Aarau, Switzerland; ^2^ Medical Faculty, University of Zurich, Zurich, Switzerland; ^3^ Institute of Pathology, Kantonsspital Aarau, Aarau, Switzerland; ^4^ Medical Faculty, University of Basel, Basel, Switzerland; ^5^ Department of Gastroenterology, Kantonsspital Aarau, Aarau, Switzerland

**Keywords:** newborn, oesophagitis, inflammatory bowel diseases, candida, case report

## Abstract

Neonatal diagnosis of inflammatory bowel disease (IBD) proves challenging due to its non-specific symptoms. A term-born neonate showing states of inflammation and aphthae was treated for sepsis and candidiasis before being diagnosed with interleukin-10 receptor deficiency and consecutive IBD. The patient was finally successfully treated by stem cell transplantation. The case illustrates the difficulties of the diagnostic course in IBD as it may mimic other diseases and emphasizes the importance of considering rare differential diagnoses early in the diagnostic process.

## 1 Introduction

Very early-onset inflammatory bowel disease (VEO-IBD) is defined as a chronic inflammatory disorder caused by dysregulated mucosal immune response with an onset within the first 6 years of life (1). An onset of the disease under the age of 2 years is often called infantile IBD. Those patients often experience a more severe disease course and are more likely to experience a monogenic variant of the disease (2). Only approximately 1% of patients had a definitive IBD diagnosis before 1 year of age (3). Clinical symptoms of VEO-IBD are often non-specific (3). This case presents a neonate that initially showed signs of general bacterial and fungal mucocutaneous infection that was later diagnosed as VEO-IBD. Genetic assessment revealed an interleukin-10 receptor (IL-10 receptor) deficiency due to a homozygote IL-10 receptor gene mutation (IL10RA, c.133T>G, p.Trp45Gly).

## 2 Case description

A female neonate was born at term at a gestational age of 40 5/7 weeks, birth weight 3,890 g, 79th percentile, and a normal Apgar score of 6/8/9 and umbilical cord pH of 7.15.

The patient was born from meconium-stained amniotic liquid at a peripheral hospital. She was the first child of distantly related parents—the child's maternal grandfather was a cousin of the child’s paternal grandmother. The patient was referred to a tertiary care neonatal intensive care unit for primary respiratory distress syndrome due to meconium aspiration and suspicion of early-onset sepsis. Body temperature was 38.8°C and treatment with amoxicillin and gentamicin was initiated after blood cultures were drawn.

After 23 h, C-reactive protein (CRP) rose to a maximum of 64 mg/L and body temperatures normalized. As blood cultures remained negative after 48 h, gentamicin therapy was ended and amoxicillin monotherapy continued for a total of 5 days with an improved general condition and at a decreased CRP of 7.5 mg/L. In general, decisions to end antibiotic therapy were based on clinical improvement, negative results from cultures, and serial CRP measurements with low values, as the latter results in a high negative predictive value (4).

**Figure 1:**
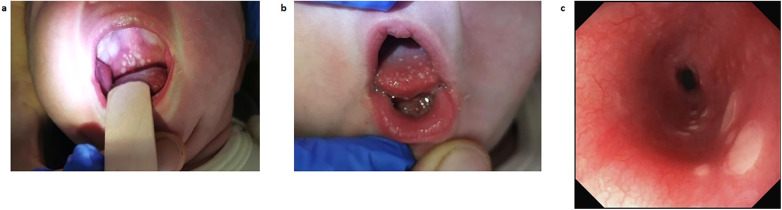
Gastroesophageal lesions as seen by inspection and on esophagogastroendoscopy on DOL 33. **(a)** Photography of the patient's palate, showing thrush-like plaque on DOL 13. **(b)** Photography of the patient's tongue, showing thrush-like plaque on DOL 13. **(c)** View of the esophageal mucosa as seen on esophagogastroendoscopy on DOL 33. DOL, day of life.

On day of life (DOL) 11, oral aphthae appeared (Figures 1a,b). The child became increasingly irritable and fever flared up for a second time (38.1°C). Diagnostics for infectious diseases, including lumbar puncture, were carried out on DOL 12 and CRP rose to 33.9 mg/L. A second course of amoxicillin and gentamicin was administered for suspected late-onset sepsis and acyclovir initiated as herpes simplex infection of the central nervous system could not be ruled out. Spinal fluid, blood culture, stool tests, and pharyngeal swab remained negative for all examined bacteria and viruses. Within 4 days, the patient’s general condition improved and CRP decreased to 20.3 mg/L; therefore, anti-infectious therapy was ended. Nevertheless, oral aphthae increased.

On DOL 16, fever flared up for a third time (38.8°C), accompanied by increasing irritability and a CRP of 17.2 mg/L. Amoxicillin and gentamicin therapy was initiated again for suspected late-onset sepsis for 6 days. Neither blood nor urine cultures nor stool tests could detect any relevant germ. Aphthae improved under therapy, body temperatures normalized, and the patient’s general condition improved. Due to a new onset of surge-like vomiting, pyloric stenosis was ruled out using abdominal ultrasound.

**Figure 2:**
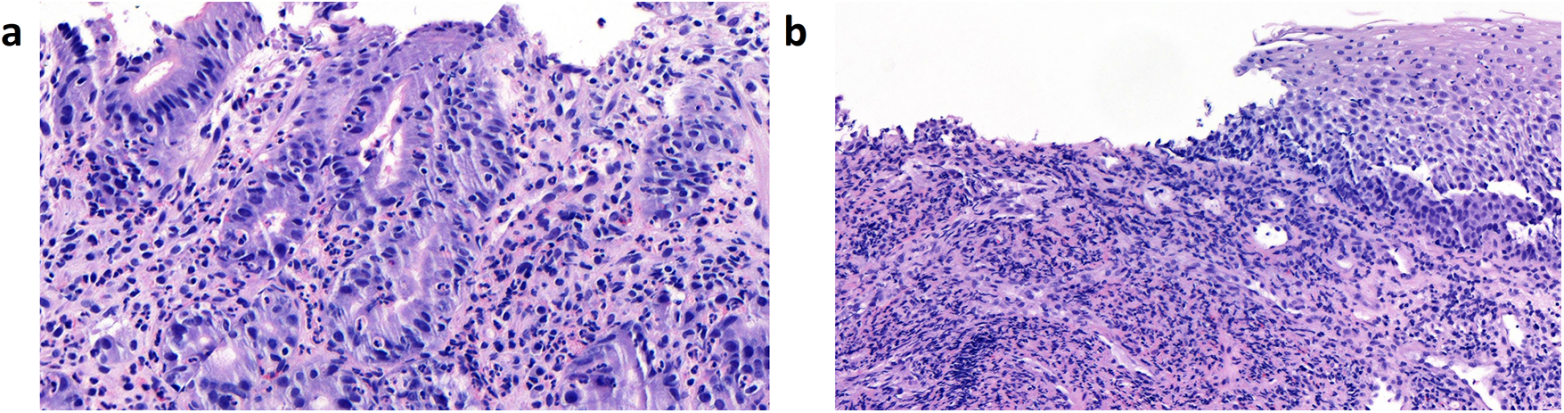
Histological images from esophagogastroendoscopy on DOL 33. **(a)** Images of mucosa from the stomach, showing an active gastritis with a mixed inflammatory infiltrate of neutrophil granulocytes and some lymphocytes. **(b)** Images of the esophageal mucosa showing a florid erosive esophagitis without evidence of fungal involvement. The inflammatory infiltrate consists predominantly of neutrophil granulocytes. Granulomas are not present. DOL, day of life.

On DOL 27, oral thrush-like efflorescence appeared that was accompanied by severe dysphagia. Reflux esophagitis and oral–esophageal candidiasis were suggested. Esomeprazole and an oral treatment with miconazole over 8 days were therefore initiated. Esophagogastroduodenoscopy was performed on DOL 33. Distal esophageal mucosa showed non-removable whitish plaques (Figure 1c), while mucosa in the stomach and duodenum appeared to be normal. Histology of the stomach revealed an active gastritis with a mixed inflammatory infiltrate (Figure 2a). No granulomas or other signs for any specific inflammatory condition were present. The esophagus showed a florid erosive esophagitis without evidence for a fungal involvement (Figure 2b). Immunohistochemically, neither *Helicobacter pylori*, nor cytomegalovirus, varicella zoster virus, or herpes simplex virus 1 or 2 could be detected.

Even though there was no evidence for fungal involvement, a systemic antifungal therapy with fluconazole was administered for a total of 4 days after esophagogastroduodenoscopy. This is because the visible non-removable whitish plaques were considered to be clinical signs for candidiasis and the lack of *Candida* in the biopsies was explained by prior oral antifungal therapy with miconazole.

On DOL 32, a fourth episode of fever (38.0°C) began. The patient showed a reduced general condition and prolonged capillary refill time. Work-up, including lumbar puncture, was repeated. Amoxicillin and gentamicin were initiated for suspected late-onset sepsis. CRP rose to 13.8 mg/L, but nasopharyngeal secretions and blood and liquor cultures were negative. As the patient’s general condition improved, antibiotic therapy was stopped after 3 days.

On DOL 40, the child was transferred to a gastroenterological and immunological referral center for suspicion of immune deficiency. Further immunological diagnostics showed non-specific altered values in the form of slightly activated clusters of differentiation 4 (CD4)+ T cells (CD3+ T cells 4.88 × 10^3 ^/µl, CD4+ T cells 4.11 × 10^3 ^/µl, CD4+ human leukocyte antigen (HLA)-DR+ CD3+ stimulated T cells 0.29 × 10^3 ^/µl, CD8+ T cells 0.63 × 10^3 ^/µl, CD19+ B cells 1.25 × 10^3 ^/µl, CD16+ CD56+ natural killer cells 0.84 × 10^3^ /µl) and decreased immunoglobulins (IgG min. 2.84 g/L, IgM min. 0.19 g/L, IgA min. 0.22 g/L) in the plasma, therefore also making a severe combined immunodeficiency unlikely. After further negative radiological, gastrointestinal and immunological diagnostics, whole genome sequencing revealed a homozygote IL-10 receptor gene mutation (IL10RA, c.133T>G, p.Trp45Gly). This is known to be a possible cause for VEO-IBD (5). A trial therapy with oral steroids was begun on DOL 63. There was no complete regression as the patient presented with episodes of bloody stools and abdominal pain. In addition, calprotectin levels were repeatedly significantly elevated, indicating that remission had not been achieved. Therefore, infliximab was administered on DOL 99 and 113. Afterwards, the patient could be fed enterally and developed interest in solid foods from the third month of life onwards with good weight gain. A prophylaxis against *Pneumocystis jirovecii* was initiated on DOL 95. She received three doses of immunoglobulins (0.4 g/kg/dose) to counteract immunosuppression and intestinal loss. The parents consented to the only curative therapy of the IL-10 receptor defect, which was stem cell transplantation at the age of 6 months: after intestinal decontamination with colistin, vancomycin, gentamicin, and amphomoronal over 10 days, conditioning with busulfan, fludarabine, and alemtuzumab over 5 days, and premedication with clemastin and prednisolone, the uneventful allogeneic hematopoietic stem cell transplantation from a matched unrelated donor was performed with two transfusion fractions (13,5 × 10^6^ CD34+/kg and 14.4 × 10^6^ CD3+/kg) on DOL 172. A graft versus host disease prophylaxis was established with ciclosporin and mycophenolate. She was discharged on DOL 220 in good clinical condition.

## 3 Timeline

**Figure 3:**
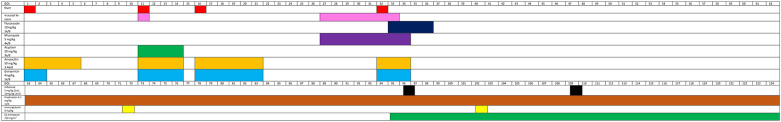
Timeline of medication of the patient and her symptoms. While the timeline focuses on the anti-infective treatment between DOL 1 and 62, the anti-inflammatory and IBD-related treatment is visualized from DOL 63. DOL, day of life; IBD, inflammatory bowel disease.

Figure 3 shows the timeline.

## 4 Therapeutic assessment

The patient was treated for suspicion of early and late-onset sepsis four times, and the hypothesis of an infectiological cause of the symptoms remained leading for almost 5 weeks of treatment even though no germ was ever detected.

First doubts about an infectiological reason arose on DOL 16, when surge-like vomiting appeared, and an ultrasound scan was performed to rule out pyloric stenosis. The decision to perform an esophagogastroendoscopy was taken as a result of dysphagia that began on DOL 27 under suspicion of possible reflux esophagitis or disseminated *Candida* infection. The lack of fungal involvement was explained by prior antifungal therapy at the time even though the histological findings, such as active gastritis and erosive esophagitis, were already in line with VEO-IBD. Only when it became clearer that the symptoms appeared and disappeared independently from anti-infective treatment did an immunodeficiency become a leading differential diagnosis, which finally led to the hospital transfer and establishment of the VEO-IBD diagnosis in the patient.

## 5 Discussion

VEO-IBD is defined as IBD occurring in the first 6 years of life (3, 6). The prevalence of VEO-IBD worldwide is in the range of 1.9–5.8 cases per 100,000 (7). VEO-IBD includes both true pediatric (polygenic) IBDs, which are Crohn's disease, ulcerative colitis, and mono- or oligogenic pathologies related to primary immunodeficiencies (8). The severity of VEO-IBD is aggravated compared to those with pediatric IBD. The response to immunosuppressants is poorer and more surgical procedures are required (9).

In the case presented, VEO-IBD was associated with an IL-10 receptor mutation. The IL-10 receptor plays a crucial role in regulating immune responses by mediating the anti-inflammatory effects of IL-10. Genetic deficiencies in the IL-10 receptor, such as mutations leading to a loss of function, can disrupt this regulatory pathway, thereby increasing the risk of developing severe immunological diseases, including IBD. A c.T133G, p.Trp45Gly mutation in the IL-10 receptor was found to be associated with early-onset IBD (10). This mutation leads to a defective receptor, impairing the IL-10 signaling pathway and thus contributing to chronic inflammation and autoimmunity.

While IBD is rare in infants, its onset within the first few months of life should raise suspicion of a genetic form of the disease. Red flags for VEO-IBD include severe, refractory gastrointestinal symptoms that do not respond to standard treatments, further failure to thrive, and perianal fistulas or abscesses. Atypical infection history, particularly recurrent or unusual infections, lymphoid organ abnormalities or tumors can indicate an underlying immunodeficiency (11). A family history of early-onset IBD as well as autoimmune disorders or consanguinity further supports a genetic cause (12). In addition, a poor response to conventional IBD therapies and laboratory evidence of immune dysregulation are strong indicators of a potential genetic etiology (13).

Our patient experienced recurrent states of inflammation and aphthae without showing any of the red flags described above apart from her two parents being distantly related. This is in contrast to other case reports of VEO-IBD in the neonatal period, where patients presented with more marked intestinal symptoms such as bloody diarrhea (14) or intractable colitis (15).

Initially, different infectiological diagnoses were considered and treated. This included late-onset sepsis, oral and esophageal candidiasis, and herpes simplex infection. Other infectiological differential diagnoses, such as Coxsackievirus infection or congenital syphilis, were considered and ruled out as negative. Because of the seemingly typical infectious symptoms, the patient received several cycles of anti-infective treatment. However, after diagnosing VEO-IBD caused by interleukin-10 receptor deficiency, all episodes were finally attributable to autoinflammatory activity rather than bacterial, viral, or fungal involvement. Our patient was treated with steroids, infliximab, and ultimately with stem cell transplantation. The latter is necessary in one-third of patients with VEO-IBD (16).

All in all, our case is typically atypical. Diagnosing VEO-IBD is challenging as the disease can present with a variety of non-specific clinical symptoms. Even if there are gastrointestinal symptoms, they might easily be mistaken for signs of infectious diseases that may delay the correct diagnosis and treatment even further.

## 6 Patient perspective

Considering the patient's age, it is not possible to give a patient's perspective. However, the patient's parents faced two phases with tremendous uncertainty and fear for their child. The first occurred before the final VEO-IBD diagnosis, with multiple infectious diagnostic tests and unsuccessful anti-infective treatments. The second phase was after the diagnosis and in the period leading up to and during the hematopoietic stem cell transplantation. The situation required very close multidisciplinary communication with the parents, including weekly discussions and updates. During the first episode, the parents received several demoralizing telephone calls in the evening or at night informing them that their child was sick again and that sepsis could not be ruled out. Every call and piece of worrying information triggered psychological and emotional distress for the parents. They managed to cope better when the steroid and infliximab treatment signs of success and the pain management became effective. Both parents adapted to the situation, and after the child was hospitalized for nearly 6 months, they were able to accept the necessary isolation precautions during stem cell transplantation, which they described as significant constraints. Finally, the success of the stem cell transplantation and the impending hospital discharge of their child raised hope and confidence in the parents, leading them to request that the intervals between parental discussions be extended to every 2 weeks.

## Data Availability

The original contributions presented in the study are included in the article/Supplementary Material, further inquiries can be directed to the corresponding author.
